# Construction of a Linked Data Set of COVID-19 Knowledge Graphs: Development and Applications

**DOI:** 10.2196/37215

**Published:** 2022-05-13

**Authors:** Haofen Wang, Huifang Du, Guilin Qi, Huajun Chen, Wei Hu, Zhuo Chen

**Affiliations:** 1 College of Design and Innovation Tongji University Shanghai China; 2 School of Computer Science and Engineering Southeast University Nanjing China; 3 College of Computer Science and Technology Zhejiang University Hangzhou China; 4 National Institute of Healthcare Data Science Nanjing University Nanjing China

**Keywords:** knowledge graph, linked data, COVID-19, knowledge extraction, knowledge fusion, natural language processing, artificial intelligence, data set, schema modeling, semantic search

## Abstract

**Background:**

With the continuous spread of COVID-19, information about the worldwide pandemic is exploding. Therefore, it is necessary and significant to organize such a large amount of information. As the key branch of artificial intelligence, a knowledge graph (KG) is helpful to structure, reason, and understand data.

**Objective:**

To improve the utilization value of the information and effectively aid researchers to combat COVID-19, we have constructed and successively released a unified linked data set named OpenKG-COVID19, which is one of the largest existing KGs related to COVID-19. OpenKG-COVID19 includes 10 interlinked COVID-19 subgraphs covering the topics of encyclopedia, concept, medical, research, event, health, epidemiology, goods, prevention, and character.

**Methods:**

In this paper, we introduce the key techniques exploited in building COVID-19 KGs in a top-down manner. First, the schema of the modeling process for each KG in OpenKG-COVID19 is described. Second, we propose different methods for extracting knowledge from open government sites, professional texts, public domain–specific sources, and public encyclopedia sites. The curated 10 COVID-19 KGs are further linked together at both the schema and data levels. In addition, we present the naming convention for OpenKG-COVID19.

**Results:**

OpenKG-COVID19 has more than 2572 concepts, 329,600 entities, 513 properties, and 2,687,329 facts, and the data set will be updated continuously. Each COVID-19 KG was evaluated, and the average precision was found to be above 93%. We have developed search and browse interfaces and a SPARQL endpoint to improve user access. Possible intelligent applications based on OpenKG-COVID19 for further development are also described.

**Conclusions:**

A KG is useful for intelligent question-answering, semantic searches, recommendation systems, visualization analysis, and decision-making support. Research related to COVID-19, biomedicine, and many other communities can benefit from OpenKG-COVID19. Furthermore, the 10 KGs will be continuously updated to ensure that the public will have access to sufficient and up-to-date knowledge.

## Introduction

On February 11, 2020, the World Health Organization announced the official name of the 2019 novel coronavirus as COVID-19. Meanwhile, the International Committee on Taxonomy of Viruses named this novel coronavirus SARS-CoV-2 [1]. The infection caused by SARS-CoV-2 is now affecting almost every country in the world. By October 24, 2021, more than 4.95 million people have died from COVID-19, raising concerns of widespread fear and increasing anxiety in individuals. At present, the epidemic continues to spread, and there are many questions that continue to plague the public about this disease, including: How can we obtain an overall understanding of the knowledge about COVID-19 facing such large amounts of information coming from various media every day? What are the variants of SARS-CoV-2 and how should they be treated or prevented? What is the state of supplies, hot events, and frontline health care workers in this invisible war worldwide? How can we find drugs or vaccines, and further learn more? What travel restrictions do local policies apply during the epidemic? What are the requirements regarding the various means of transport?

During this pandemic, artificial intelligence (AI) has served as an enabler to combat COVID-19, such as successful attempts in predicting epidemic trends [2] with sophisticated models, accelerating computer tomography detection [3] for more efficient diagnosis by computer vision, participating in drug development [4], and automatically answering epidemic-related natural language questions [5-7]. Besides deep learning, the knowledge graph (KG) concept has drawn increasing attention from both academia and industry since it was first proposed by Google in 2012. As the key to the evolution of AI toward cognitive intelligence, a KG enables machines to better organize, reason, understand, and explain information.

The success of the above applications heavily depends on the scale and quality of the underlying KGs, regardless of whether they exist in the open or in a specific domain. Well-known general-purpose KGs include DBpedia [8], Yago [9], Freebase [10], Wikidata [11], and the Chinese linked open data effort Zhishi.me [12]. All of these KGs leverage Wikipedia, one of the largest encyclopedia websites in the world, as an important source. WordNet [13], BabelNet [14], and Linguistic Linked Open Data [15] are examples of linguistic KGs. Regarding domain-specific KGs, we here mainly focus on life science or health care fields. The KG Linking Open Drug Data [16] surveys the publicly available data about drugs and creates linked representations of the data sets. The project Open PHACTS [17] aims to deliver and sustain an open pharmacological space using and enhancing state-of-the-art semantic web standards and technologies. Bio2RDF [18] uses semantic web technologies to provide the largest network of linked data about the life sciences. However, none of the above KGs is specific to COVID-19. Although it is possible to extract a COVID-19–relevant subgraph from general-purpose KGs, this approach will suffer from low coverage of domain knowledge and the sparsity of properties describing this knowledge (eg, viruses and diseases).

The White House, in collaboration with publishers and tech firms, has launched the CORD-19 data set [19], which contains more than 59,000 published articles and preprints. Although CORD-19 is considered to be the largest single collection of COVID-19 knowledge amassed to date, the majority of the data set contains unstructured data, and more than 60% of the included papers do not mention search terms such as “coronavirus” and “SARS-CoV” [20]. The existing COVID-19 Knowledge Graph [21] is an expansive cause-and-effect network constructed from the scientific literature on SARS-CoV-2, aiming to provide a comprehensive view of its pathophysiology. However, there are only 10 entity types and 9484 facts within this KG. Coronavirus Knowledge Graph [22] only has 27 relation types. The CovidGraph project [23] built a COVID-19 graph that stores publications, case statistics, and molecular data in a Neo4j database, which enables exploring the underlying knowledge for finding specific genes, authors, articles, patents, proteins, existing treatments, and medications relevant to the entire family of coronaviruses. However, key aspects such as health care, epidemiology, antiepidemic goods, related events, and frontline workers fighting the epidemic have not yet been considered.

**Figure 1:**
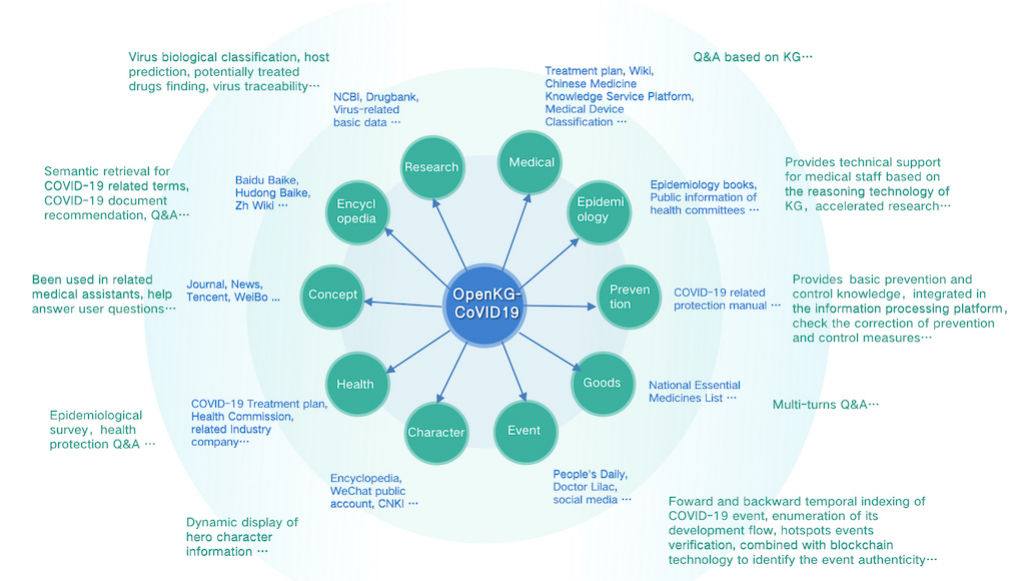
Overview of OpenKG-COVID19. KG: knowledge graph; NCBI: National Center for Biotechnology Information; Q&A: question and answer.

To capture richer and more diverse topics of COVID-19 so as to offer more useful knowledge for the public, we have extended these previous efforts [24-26] to construct OpenKG-COVID19, a linked data set of COVID-19 KGs, covering 10 aspects ranging from encyclopedia, concept, medical, health, prevention, goods, research, epidemiology, and character to events. OpenKG-COVID19 was launched by OpenKG [27], which is the largest Chinese open KG community pushing for the development of public KGs, open-source tools, and best practices in vertical sectors in China since the middle of February 2020. We are the first to mainly focus on constructing high-quality pandemic KGs in China. Moreover, OpenKG-COVID19 is open to the public with continuous efforts to ensure that it contains up-to-date information. The publishing and maintenance of such a large-scale KG can help researchers around the world to understand, study, and even fight COVID-19. An overview of OpenKG-COVID19 is depicted in Figure 1. Each KG, its sources, and possible applications are listed in Textbox 1.

Moreover, several key steps have been used to construct OpenKG-COVID19, namely modeling, extraction, and fusion of knowledge. Among them, knowledge modeling mainly involves schema design. The schema knowledge of each data set in OpenKG-COVID19 is described in the Methods section. The other steps are executed automatically with the human in the loop. In particular, we present the technical details of knowledge extraction and then describe how the curated KGs are further linked together at both the schema and data levels. We further present the results of experimental validation of OpenKG-COVID19, and discuss the access interfaces along with the possible applications of the linked COVID19 KGs.

**Sources, knowledge graphs (KGs), and application prospects of OpenKG-COVID19.**

- The encyclopedia KG (Bilingual Encyclopedia Knowledge Graph [BEKG]) is based on multiple encyclopedia sources, which helps to gain a basic understanding of SARS-CoV-2 and COVID-19.
- Targeting the question and answer (QA) system, both the medical KG and the health KG consider data sources from industrial companies and official treatment plans, which have included COVID-19–related symptoms, diseases, drugs, and treatment options.
- The prevention KG not only provides authoritative guidance on individuals’ protection and public prevention, but also contains knowledge about vaccines and nucleic acid tests.
- The goods KG provides the current status of materials used in the epidemic, including information of daily protective equipment, medical diagnosis, treatment devices, and therapeutic drugs.
- The research KG aims to assist in the discovery of drugs or vaccines, and its data are derived from virus-related scientific research databases and literature.
- The epidemiology KG helps to trace the source of infection and explore contacts. These data come from the case flow information published by provincial health committees.
- The character KG sorts out heroic deeds and assists in the dynamic display of character information, including the individual’s resume, achievements, and related events about combating the epidemic.
- The event KG organizes hot events about the epidemic with the when, where, who, and what factors incorporated.
- The concept KG uses automatic web-mining technologies to collect a large number of fine-grained COVID-19–related entities and their corresponding hypernyms from web text, which has been applied in medical-related virtual assistants to address complex user information needs.

## Methods

### Schema of OpenKG-COVID19

A schema defines a specific, clear, high-level structure of a KG. It is necessary to model a sound schema to accurately offer a clear understanding of KG content. New data added to the KG will not be allowed if the data do not conform to the defined schema. We designed a total of 10 schemata for each subgraph: concept, encyclopedia, medical, health, research, prevention, goods, event, character, and epidemiology. The details of the schemata are described in further detail elsewhere [28]. In brief, three methods were employed to develop the schemata: manually defined by medical experts (manual), extracted from encyclopedic websites or COVID-19–related medical websites (site data), and mined automatically from the web (automatic mining). The design method of each KG is displayed in the left part of Table 1.

**Figure 2:**
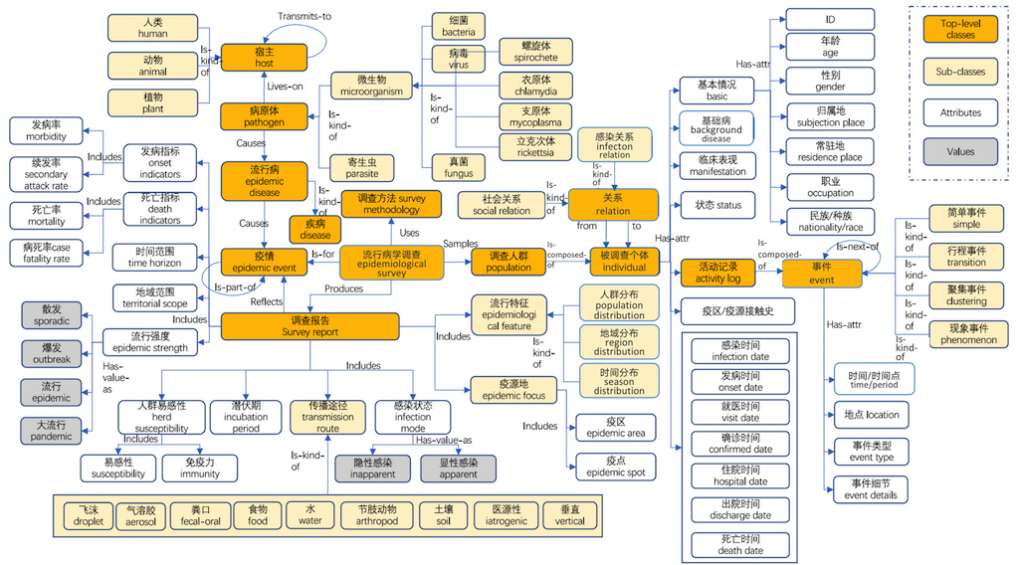
Schema diagram of the epidemiology knowledge graph.

Within OpenKG-COVID19, the schemata of most KGs (eg, medical, epidemiology) have been designed by domain experts. Taking the epidemiology KG as an example, its schema defines the basic concepts of epidemiology such as epidemic, pathogen, host, epidemic situation, epidemiological survey, survey method, survey population, surveyed individual, and survey report. The relations between these concepts contain “cause,” “is-part-of,” “includes,” “uses,” and similar. The entire schema diagram is illustrated in Figure 2. Note that even though the schema shown here was manually constructed, we can boost the entire process by recommending users’ domain keywords or related ontologies in the same or similar field as a prototype for reuse.

**Figure 3:**
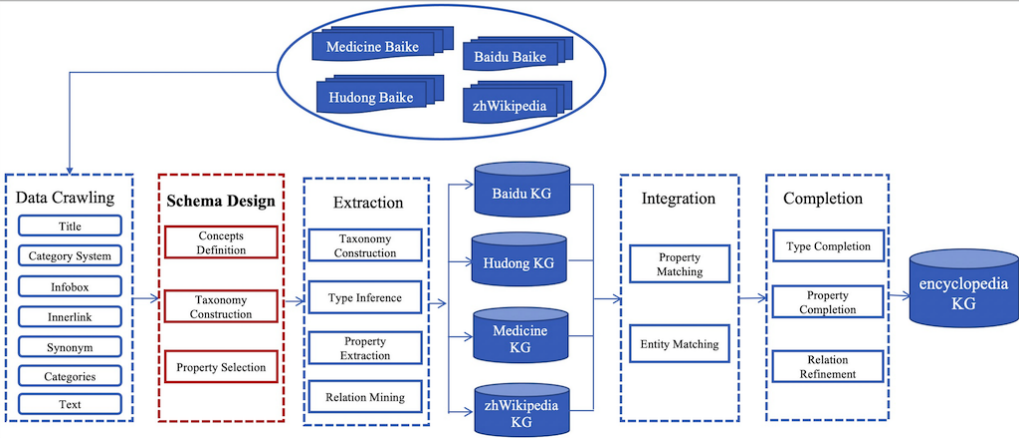
Construction process of the encyclopedia knowledge graph (KG).

Another method for schemata design is to treat semistructured information as categories and properties in “infoboxes” as schemata. This method was used for the design of the schemata for the encyclopedia and research KGs. Specifically, the schema modeling process during construction of the encyclopedia KG is shown with a red color border in Figure 3. We further used BabelNet [29] and Zhishi.schema [30] to expand the concepts with multilingual labels.

We also tried to automatically mine schemata from the web. Specifically, we performed nonlinear mapping between one concept to another (its hypernym) based on popular embedding technology to obtain a large number of fine-grained hypernyms from search engines, encyclopedias, and word morphology. The hierarchical structure (“is-a” relation) was constructed by measuring the semantic broadness between concepts as well as between an instance and a concept. Therefore, the data-level knowledge was also extracted during schema design.

**Table 1:** Classifications of schema design and knowledge extraction of COVID-19 knowledge graphs.

Knowledge graph	Schema design				Knowledge extraction		
	Manual	Site data	Automatic mining	Structured		Semistructured	Plain text
Concept			✓			✓	✓
Encyclopedia		✓	✓			✓	✓
Medical	✓						✓
Health	✓	✓				✓	
Research		✓		✓			✓
Prevention	✓			✓		✓	✓
Goods		✓				✓	
Event	✓						✓
Character		✓				✓	
Epidemiology	✓			✓			

### Knowledge Extraction for COVID-19 KGs Construction

#### Overview

This section introduces methods for the classification of knowledge extraction based on different data types. In general, sources of knowledge extraction include structured data (eg, linked data), semistructured data (eg, tables and infoboxes), and unstructured data in the form of plain text. Sources of each KG in OpenKG-COVID19 are listed in the right part of Table 1. There exist correlations between schema design and sources for knowledge extraction. For example, if a KG extracts its knowledge from semistructured data sources, its schema is usually obtained from site data. Graph mapping is leveraged to extract a domain-specific subgraph from linked data, whereas the “D2R” tool is used to transform relational data of a Database into Resource Description Framework (RDF) triples. Moreover, wrappers are used for semistructured data and information extraction to convert plain text into structured knowledge.

#### Extraction from Structured Data Sources

Structured data represent the main data source of KG construction. Our research KG focuses on information from the virus field. It contains five subgraphs, which are the virus taxonomy KG, SARS-CoV-2 gene-protein KG, antiviral drug KG, SARS-CoV-2 phylogeny KG, and SARS-CoV-2 literature extraction KG. The construction of the first four KGs fits within this method.

Specifically, we analyzed some data of related biodatabases (eg, National Center for Biotechnology Information [NCBI] [31], GISAID [32], China National Center for Bioinformation [33], DrugBank [34], and Nextstrain [35]) and related biological KGs such as SNAP [36] at Stanford University. Moreover, we have established in-depth collaborations with some biological institutes in the vertical field to ensure that the research KG is professional. We converted data in different formats from the above sources into a unified graph structure based on the designed schema.

The SARS-CoV-2 gene-protein KG is mainly built from the virus data in the NCBI database. By looking up “SARS-CoV-2” in NCBI, various types of related information are returned, such as genome, gene, and protein. Two example triples are (SARS-CoV-2, Virus-express-Gene, NS6) and (SARS-CoV-2, Virus-produce-Protein, nonstructural protein NS6).

The antiviral drug KG is based on four structured databases: DrugBank, Virus Pathogen Database [37], VirHostNet 3.0 [38], and VISDB [39]. The KG demonstrates interaction relationships among various types of viruses, human proteins, antiviral drugs, and diseases. For further integration, we linked the data through the taxonomy ID of the virus, the UniProt ID of the protein, and the generic name of the drug. Several extracted example triples are: (Human immunodeficiency virus 1, Virus-alias-String, HIV-1), (Enfuvirtide, Drug-effect-Virus, Human immunodeficiency virus 1), and (H31, HostProtein-belong to-Host, Human).

We also extracted the virus taxonomy tree from NCBI to build the corresponding KG. Similarly, the SARS-CoV-2 phylogeny KG was constructed by referencing Nextstrain metadata.

#### Extraction From Semistructured Sites

**Figure 4:**
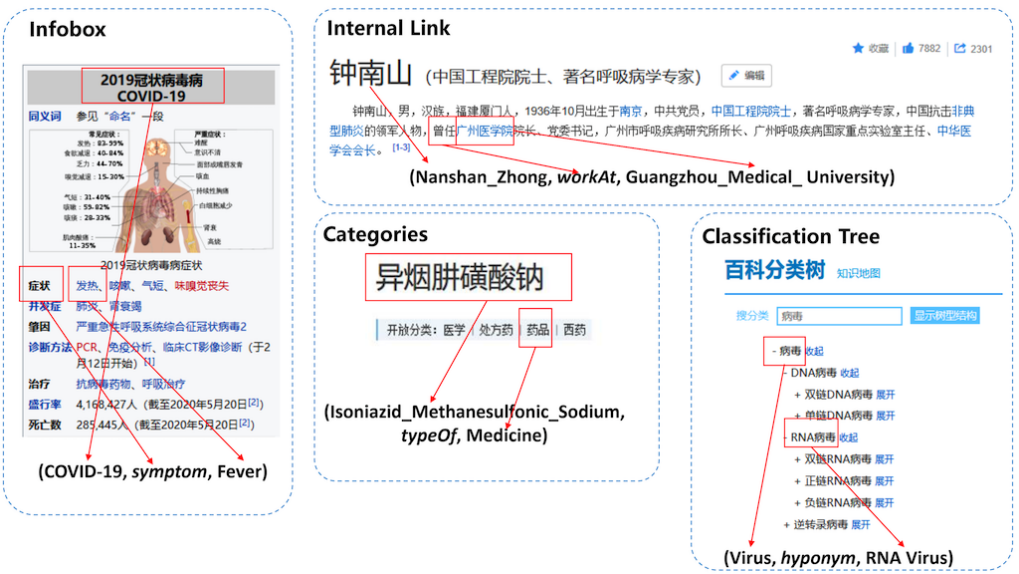
Extraction of various types of semistructured data.

We mainly leveraged semistructured data for building the KGs of concept, encyclopedia, health, prevention, goods, and character. Taking the encyclopedia KG as an example, its knowledge in the form of RDF triples is extracted from the integration of several encyclopedia sites (eg, Baidu Baike, Hudong Baike, Chinese Wikipedia). We particularly considered the following four types of semistructured data for knowledge extraction: internal links, infoboxes, categories, and classification trees. For an infobox, the page title is treated as a subject, each attribute of the infobox is treated as a predicate, and the corresponding attribute value is treated as the object. For an internal link, we also treat the title entity as a subject, the target entity that the internal link refers to as the object, and the relation (defined in the schema) matching the text between the subject mention and the object mention as the predicate. For a category that a page belongs to, the title entity, typeOf, and the given category form a triple. For a classification tree, a high-level concept can be linked with any of its ancestors in a triple using the hyponym as the predicate. Figure 4 shows examples for all four types of semistructured data and their corresponding extracted triples.

#### Extraction From Plain Texts

Plain texts are widely available for human consumption but are hard for machines to understand, which hinders construction of a KG from these unstructured data. We applied regular expressions to extract fact triples for the six subgraphs shown in the right part of Table 1 from plain texts. When detecting knowledge by regular expressions, we paid more attention to the precision of information extraction rather than the recall to ensure that the COVID-19 knowledge managed into OpenKG-COVID19 is relatively accurate. Finally, the average precision of our regex matching methods was found to be 96.34% and the average recall was 87.63%. However, there are large amounts of diverse information and complex semantic relations in the research literature, which required more advanced methods during the construction of the research KG. In recent years, there has been great progress in applying machine reading comprehension to the knowledge extraction task on plain texts [40,41]. The basic idea is to extract the candidate entities from sentences by a subject extraction network, and then extract the object of a triple based on candidate entities and a predefined predicate using a joint predicate-object extraction network. Pretrained language models such as bidirectional encoder representations from transformers (BERT) [42] are employed for encoding in both networks, which alleviates the amount of labeled data required to train a model.

Inspired by the above work, we applied the same technique in building COVID-19 KGs from various text sources. The labeling process can be further relieved by distant supervision [43], where the subject and object of a triple are automatically labeled in one sentence and the sentence context is captured to check whether the predicate holds. After extraction and sampled manual check, triples such as (SARS-CoV2, Virus-interaction-Human Protein, ACE2), (SARSCoV-2, Virus-cause-Disease, human respiratory disease), and (nelfinavir, Drug-effect-Virus, SARS-CoV2) are returned from the medical literature.

### Interlinking Knowledge from Different COVID-19 KGs

#### Overview

Following the linked data principles, we connected these KGs to promote the integration and sharing of knowledge about COVID-19. We observed that schemata in these KGs, except for that of the concept KG, are of relatively small scale. Therefore, we first used an automatic ontology matching approach to align schema-level knowledge (ie, concepts and properties) and then asked domain experts to validate the results, and finally leveraged the validated schema matches to align data-level knowledge (ie, entities).

#### Schema Matching

Because there is no central schema for the COVID-19 KGs, we decided to conduct pairwise schema matching. We reused Falcon-AO [44], which is an automatic ontology matching tool. Its main strengths lie in the integration of various powerful matchers exploiting linguistic and structural features. Furthermore, due to the naming issue, many schemata use sequential IDs to name their concepts and properties. To avoid their interference with the matching process, we disabled the comparison of local names in Falcon-AO. The details of the naming convention are introduced below.

#### Entity Alignment

**Figure 5:**
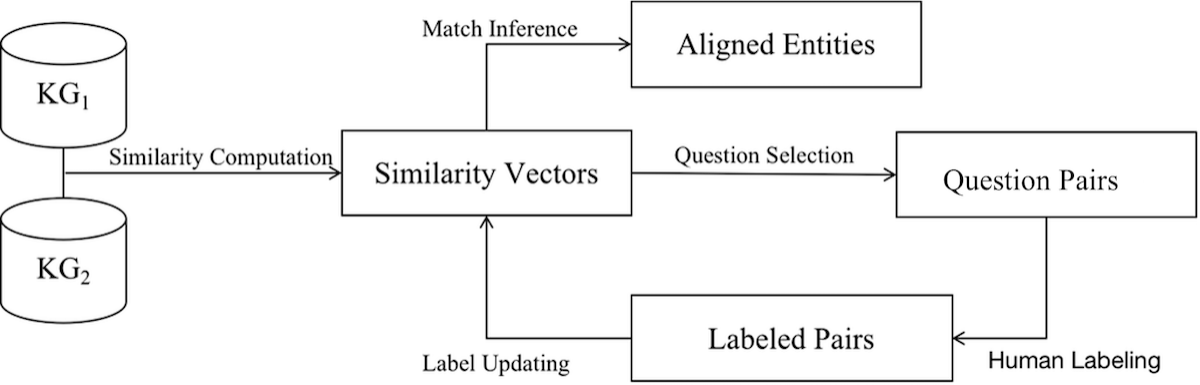
Workflow of entity alignment.KG: Knowledge graph.

Similar to schema matching, we conducted pairwise entity alignment. We used the property matches from schema matching to make the properties in each pair of KGs uniform. We observed that most matched properties are data-type properties. Therefore, we leveraged literal similarity measures to align entities. Since the true matches are unavailable, we deployed the crowdsourced entity resolution approach [45] to find entity matches, and the workflow is depicted in Figure 5.

#### Similarity Computation

For each entity pair, we used similarity measures to construct a similarity vector, where each real value in the vector represents the similarity of the values of each pair of aligned properties. For numerical values, the absolute difference similarity measure was used. For textual values, the Jaccard similarity measure was applied. Moreover, the entity-type values were converted to texts based on their labels. Note that a few KGs are multilingual; therefore, we used a character-level bigram to tokenize textual values.

#### Match Inference

Based on similarity vectors of entity pairs, we used the partial order assumption to infer matches and nonmatches. Once an entity pair is judged as a match by a human, each entity pair such that all similarity values are not less than those of the match is inferred as a match. By contrast, once an entity pair is judged as a nonmatch, each entity pair such that all similarity values are not greater than those of the nonmatch is inferred as a nonmatch. When the similarity measures evaluate the value within the threshold range, these inference rules are approximately true [46].

#### Question Selection

To save both human labor and time, the total number of questions (ie, unresolved entity pair for validation) is required to be minimized. However, the true answers for questions are unknown. Alternatively, we maximized the inference power of a new question in each step. The question-selection algorithm iteratively chooses each unresolved pair that has the greatest number of possible inferred matches and nonmatches.

#### Human Labeling

Some KGs contain a lot of medical details (eg, drugs in research, posthospital medications, limitations, special diets); thus, common workers from the crowdsourcing platforms may not have sufficient domain knowledge to manage a large amount of medical information. To ensure a data set of high quality and benefit to downstream tasks such as question answering, we employed expert sourcing instead of crowdsourcing to collect answers for questions pairs. In detail, we asked one domain expert to judge each unresolved pair as a match or a nonmatch, and randomly sampled some labeled question pairs for further review to obtain the final result.

## Results

### Data Evaluation

#### Data Statistics

OpenKG-COVID19 is a linked data set of COVID-19 KGs consisting of 10 subgraphs derived from different sources such as research publications, medical guidelines, and encyclopedia websites. As of October 24, 2021, the data set has knowledge of more than 2572 concepts, 329,600 entities, 513 properties, and 2,687,329 facts. Moreover, the data set will be updated continuously along with the occurrence of COVID-19. The detailed statistics of each KG are listed in the left part of Table 2, demonstrating that the research KG contains the largest numbers of both entities and facts, and all KGs have relatively rich properties, except for the concept KG that only defines two properties (ie, typeOf and subClassOf) but has the highest number of concepts.

**Table 2:** Detailed statistics and quality of each subgraph.

Knowledge graph	Facts, n	Concepts, n	Entities, n	Properties, n	Evaluation, n	Correct, n	Precision (%), mean (SD)
Encyclopedia	261,154	50	54,318	60	5000	4778	95*.*52 (0*.*58)
Medical	2857	54	1035	92	652	620	94*.*81 (1*.*71)
Research	2,281,797	31	221,131	64	8556	8555	99*.*96 (0*.*03)
Event	27,388	4	2291	21	200	198	96*.*35 (1*.*69)
Character	1902	21	1057	40	570	570	99*.*65 (0*.*35)
Prevention	28,651	113	34,859	24	646	630	97*.*20 (1*.*25)
Goods	3738	165	132	57	365	359	97*.*83 (1*.*42)
Health	51,575	592	7110	104	487	483	98*.*78 (0*.*91)
Epidemiology	8336	55	2163	47	200	200	98*.*08 (1*.*92)
Concept	19,391	1487	4784	2	100	96	92*.*31 (4*.*96)

#### Accuracy Evaluation

It is crucial to assess the quality of each KG in OpenKG-COVID19. Since no ground truths are available, we performed manual evaluation. Owing to the large number of facts, we adopted a similar method as that of Yago with respect to the sampling strategy and labeling process.

For sampling, we evaluated a chosen sample of facts for each property defined in OpenKG-COVID19. Since the fact number of each property is not evenly distributed, we used different sampling coefficients (ranging from 0 to 1) for different properties. If the fact number of one property is lower than the minimal sample number k (k=20 in our setting), it was set to 1. Otherwise, we selected a random coefficient to ensure that the returned samples are more than k.

For labeling, we invited three postgraduate students focusing on KGs as their main research area to review the same sampling data for each subgraph. They were offered three choices to annotate each sample: agree, disagree, and unknown. If more than one annotator made a certain choice, then the sample was labeled as that choice. If there were three different annotations for one sample, we asked the annotators to reconsider the choice through acquiring further knowledge about the sample and obtain a result. However, discrepancies only accounted for 6% of all samples according to the record of the labeling process. After the labeling process, 98.35% of the sampled facts were considered to be correct by consensus. To generalize our results on the subset to the whole data set of COVID-19 KGs, the Wilson interval at α=5% was computed.

The precision value of each COVID-19 KG is reported in the right part of Table 2. We found that all KGs achieved an average precision of more than 93%, except for the concept KG with knowledge extracted by automatic web mining, which indicates the high quality of OpenKG-COVID19. After the error analysis, we found two typical patterns of wrong facts. One is that there exists a mistake of either the head entity or the tail entity, and the other is that the relation between the entity pair does not conform to the fact. For example, it is inappropriate to regard “judgment basis” as the relation between “confirmed cases” and “shock,” because this is simply a possible clinical manifestation of patients with COVID-19.

#### Results and Quality of Interlinking

The schema matching results are shown in Table 3, demonstrating overlaps between different schemata, although such overlaps are limited. Regarding entity alignment, we found 1055 matches among five KGs. The encyclopedia KG had the greatest number of matches with other KGs (ie, 836 with the health KG, 55 with the medical KG, 11 with the character KG, and 2 with the goods KG) because it contains various types of entities (eg, drugs and hospitals). We also noted some entity matches but no schema matches between the encyclopedia KG and the character KG, because some shared properties (eg, rdfs:label) are used to align entities but these properties are not included in schema matching. We also found some duplicated entities in the encyclopedia KG because these entities are extracted from different websites. There were few matches between the goods KG and other KGs because most entities in the goods KG are medical devices, which do not appear in the other KGs. Since some entities in the character KG are hospitals, there were 19 matches with the health KG. The remaining matches were mostly related to drugs.

We recruited three students with a major in Semantic Web to evaluate the precision, recall, and F1-score of the schema matching and entity alignment results. As shown in Table 4, the schema matching achieved high recall, but relatively low precision. Most false matches were caused by the similarity measure (eg, the pair “determination of protein” and “protein” was wrongly judged as a match). We observed that the entity alignment achieved perfect results in all KG pairs except for health-character with precision, recall, and F1-score of 88.2%, 100.0%, and 93.8%, respectively. The high performance of entity alignment was attributed to the fact that the literal information in KGs is of high quality and most matches share exactly the same information.

**Table 3:** Results of schema matching.^a^

Knowledge graph	Encyclopedia	Prevention	Concept	Health	Research	Medical	Epidemiology	Event	Goods	Character
Encyclopedia	—^b^	0	0	9	4	6	1	0	0	0
Prevention	0	—	0	0	0	2	0	16	17	1
Concept	37	7	—	0	0	0	0	0	0	0
Health	9	0	41	—	3	13	3	1	1	0
Research	2	0	6	2	—	3	0	1	0	0
Medical	4	2	25	4	4	—	6	3	0	1
Epidemiology	4	0	18	3	0	3	—	0	0	4
Event	0	5	1	0	0	0	1	—	16	0
Goods	0	1	18	6	0	0	0	2	—	0
Character	0	2	11	0	5	5	3	1	0	—

^a^The numbers below the diagonal are class matches and the numbers above the diagonal are property matches.

^b^Not applicable.

**Table 4:** Performance of schema matching.

Knowledge graph	Class (%)				Property (%)		
	Precision	Recall	F1-score	Precision		Recall	F1-score
Encyclopedia	75.0	85.7	80.0	85.0		85.0	85.0
Prevention	76.5	100.0	86.7	94.4		100.0	97.1
Concept	72.0	90.1	80.0	—^a^		—	—
Health	55.4	94.7	69.9	76.7		88.5	82.1
Research	78.9	100.0	88.2	54.5		100.0	70.6
Medical	87.2	91.1	89.1	79.4		90.0	84.4
Epidemiology	78.1	100.0	87.7	78.6		100.0	88.0
Event	100.0	100.0	100.0	91.9		100.0	95.8
Goods	70.4	76.0	73.1	97.1		100.0	98.5
Character	100.0	100.0	100.0	83.3		83.3	83.3
Overall	74.6	91.5	82.2	85.6		95.0	90.0

^a^Not applicable.

### Knowledge Access, Sustainability, and Possible Applications

#### Naming Convention

For considerations of readability and interoperability, we followed the RDF naming convention, which helps to quickly locate and understand the topic and the meaning of each triple. The convention is composed of three major parts.

The first is the resource identifier, in which each resource (ie, concept, entity, property) is identified by a global ID that is an integer number prefixed by a letter. That is, classes are prefixed by C (eg, C1), entities are prefixed by R (eg, R122), and properties are prefixed by P (eg, P31). The second is the uniform resource identifier (URI) pattern. All URIs should follow a pattern such as [URL]/[graphname]/[type]/[resource], where graphname is the name of the subgraph (eg, medical, research), type takes on an enumerable value representing the URI type (ie, class, resource, property), and resource is the global identifier described in the resource identifier part. The third part is the predicate usage; the COVID-19 KGs use the set of predicates shown in Table 5 to illustrate the schema model.

**Table 5:** Primary predicates used in the OpenKG-COVID19 schemata.

Predicate	Description
rdf:label	Local name statement of all URLs
rdfs:subClassOf	The hypernym-hypernym relationship between two classes
rdfs:domain	Domain class of a property
rdfs:range	Range class or literal data types of a property, which can be multivalued
owl:sameA	Synonym relationship between two resources

#### Search and Browse Interfaces

**Figure 6:**
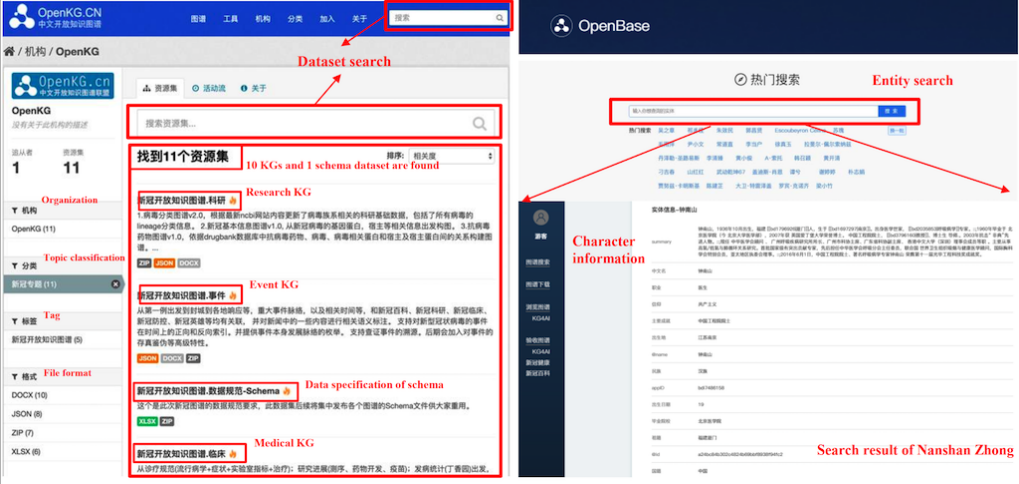
Data set search interface (left) and entity search interface (right).

Since the published KGs in OpenKG-COVID19 comply with the creative commons-by share-alike license, users can feel free to download any of them [47]. The left part of Figure 6 shows a snapshot of the data set search interface, where 10 KGs and a schema data set about OpenKG-COVID19 are found. Moreover, users can search for a particular entity and browse the detailed information of that entity in the OpenBase website [48]. As shown in the right part of Figure 6, the search results contain various properties of Nanshan Zhong, a famous doctor combating the COVID-19 epidemic in China.

#### SPARQL Endpoint

The SPARQL endpoint [49] of OpenKG-COVID19 is built upon a scalable graph database, gStore [50], which provides extendable distributed storage management as well as efficient implementations of complex queries and update operations based on SPARQL for RDF data sets with up to billions of triples. Users can submit SPARQL queries to the endpoint where relevant results are returned in the form of a table. Users can also choose to download the results packaged in a JavaScript Object Notation (JSON) file by clicking “Click to Download.” As of May 22, 2020, we have recorded over 20,000 accesses to the endpoint.

#### Sustainability and Knowledge Review

OpenKG-COVID19 KGs are maintained by the OpenKG community. We are collecting questionnaires considering users’ needs and updating our KGs accordingly. COVID-19 KGs are particularly important for timely updates because users’ needs may change as the epidemic develops (eg, from source to treatment).

**Figure 7:**
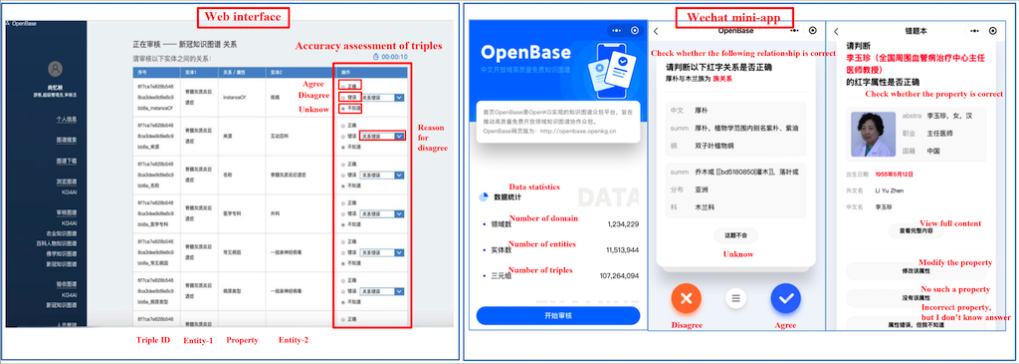
Knowledge review on the web (left) and WeChat mini app (right) of OpenBase.

The data quality as well as the interlinking quality of OpenKG-COVID19 are manually evaluated. OpenBase is a knowledge crowdsourcing platform powered by blockchain technologies for provenance tracking and credit incentive. We uploaded a part of the data that may contain errors due to the sampling method, and created many microtasks for reviewing the correctness of triples. The reviewers were volunteers certified by possession of one specific domain knowledge. They were able to not only review the KG data but to also commit data corrections. All volunteers participating in knowledge reviewing via either a web-based interface or the WeChat mini app (see Figure 7) received a corresponding reward of credit for their contributions to improving our KGs.

#### Possible Intelligent Applications

OpenKG-COVID19 is the basis of various intelligent applications, whose release will help to fight against this global plague. OpenKG-COVID19 benefits from intelligent question answering, semantic search, recommendation systems, as well as the abilities for visualization, mining more associations, predicting future events, and assisting in decision-making. More specifically, we here take the event KG, research KG, medical KG, and overall OpenKG-COVID19 as examples.

The event KG includes the forward and reverse indexing of events about COVID-19 in time, and provides the development context of a series of events, which can support the verification and traceability of hot events. Furthermore, the event KG combined with blockchain technology could identify whether or not an event is true.

Based on the research KG, Huawei Cloud has developed a personalized visual query system, displaying knowledge points and their relations, which can quickly trace the source of information and directly locate relevant documents and paragraphs. The research KG facilitates scientific research on virus mechanisms and viral protein interactions, and assists drug developers in more accurate and effective drug target research and vaccine development.

Starting from the cases of diagnosis and treatment to research progress, the medical KG is developed by extracting knowledge from the existing standard documents and the web. The epidemiology, symptoms, laboratory indicators, treatments, drug development, and vaccines of COVID-19 could be conveniently consulted making use of question answering based on the medical KG. Drugs that alleviate symptoms and potential therapeutic drugs, such as the repurposing of old drugs for a new use, can also be mined by the medical KG.

Moreover, OpenKG-COVID19 is an enabler to accelerate the development of bioinformatics. The network structures of COVID-19 KGs can be used to predict relations such as host-virus, drug-virus, or interactions between viruses and the host protein, which will help to reveal the underlying mechanism of COVID-19. In particular, the combination of protein-protein interactions, drug–protein target interactions, and the polypharmacy side effects could predict unknown side effects.

## Discussion

### Principal Results

In this study, we constructed OpenKG-COVID19, one of the largest existing KGs about COVID-19. We first presented the schema design process of OpenKG-COVID19. We then introduced the comprehensive techniques for knowledge extraction and knowledge fusion. Moreover, we provided an evaluation of the quality of OpenKG-COVID19. This paper also provides an introduction of various access interfaces covering searching, browsing, querying, and knowledge review, and discusses the possible applications of OpenKG-COVID19. Our efforts can benefit KG, biomedicine, and many other communities. New knowledge for the 10 KGs will be updated continuously through the processes described above to maintain and update OpenKG-COVID19 for improving its quality and coverage.

### Limitations

Although OpenKG-COVID19 is updated continuously, the update frequency is not daily, which may result in some information not being up to date, causing inconvenience for downstream tasks. Moreover, it is also very necessary to control the data set version, which is future work to be considered.

We randomized a chosen sample of facts for each property defined in OpenKG-COVID19 to evaluate the data quality. In some cases, the number of samples may be small, which will lead to a less reliable evaluation result. Therefore, we plan to further improve the quality of data by selecting a new method to sample more triples of each property.

### Conclusion

A KG is an effective technique to provide well-organized data, and is also beneficial for intelligent question answering, semantic search, recommendation system, visualization analysis, and decision-making support. OpenKG-COVID19 includes rich and diverse topics of COVID-19, covering 10 aspects ranging from encyclopedia, concept, medical, health, prevention, goods, research, epidemiology, and character to events. The publishing and maintenance of OpenKG-COVID19 can help researchers around the world to better understand, study, and even fight COVID-19.

## Abbreviations

AI: artificial intelligence
BERT: Bidirectional Encoder Representations from Transformer
JSON: JavaScript Object Notation
KG: Knowledge Graph
NCBI: National Center for Biotechnology Information
RDF: Resource Description Framework
URI: uniform resource identifier
